# Evolution of the Aging Brain Transcriptome and Synaptic Regulation

**DOI:** 10.1371/journal.pone.0003329

**Published:** 2008-10-02

**Authors:** Patrick M. Loerch, Tao Lu, Kelly A. Dakin, James M. Vann, Adrian Isaacs, Chengiz Geula, Jianbin Wang, Ying Pan, Dana H. Gabuzda, Cheng Li, Tomas A. Prolla, Bruce A. Yankner

**Affiliations:** 1 Department of Pathology, Harvard Medical School, Boston, Massachusetts, United States of America; 2 Department of Genetics and Medical Genetics, University of Wisconsin, Madison, Wisconsin, United States of America; 3 Cognitive Neurology and Alzheimer's Disease Center, Northwestern University, Chicago, Illinois, United States of America; 4 Department of Cancer Immunology and AIDS, Dana-Farber Cancer Institute, Harvard Medical School, Boston, Massachusetts, United States of America; 5 Department of Biostatistics, Dana-Farber Cancer Institute, Harvard School of Public Health, Boston, Massachusetts, United States of America; Temasek Life Sciences Laboratory, Singapore

## Abstract

Alzheimer's disease and other neurodegenerative disorders of aging are characterized by clinical and pathological features that are relatively specific to humans. To obtain greater insight into how brain aging has evolved, we compared age-related gene expression changes in the cortex of humans, rhesus macaques, and mice on a genome-wide scale. A small subset of gene expression changes are conserved in all three species, including robust age-dependent upregulation of the neuroprotective gene apolipoprotein D (APOD) and downregulation of the synaptic cAMP signaling gene calcium/calmodulin-dependent protein kinase IV (CAMK4). However, analysis of gene ontology and cell type localization shows that humans and rhesus macaques have diverged from mice due to a dramatic increase in age-dependent repression of neuronal genes. Many of these age-regulated neuronal genes are associated with synaptic function. Notably, genes associated with GABA-ergic inhibitory function are robustly age-downregulated in humans but not in mice at the level of both mRNA and protein. Gene downregulation was not associated with overall neuronal or synaptic loss. Thus, repression of neuronal gene expression is a prominent and recently evolved feature of brain aging in humans and rhesus macaques that may alter neural networks and contribute to age-related cognitive changes.

## Introduction

Aging is the primary risk factor for Alzheimer's disease and other prevalent neurodegenerative disorders [1], [2]. Little is known, however, about the degree to which normal brain aging is conserved among mammalian species, an issue of central importance in the biology of aging and the development of animal models of human neurological diseases [3]. Gene expression changes that appear during normal brain aging have been explored using microarrays that interrogate only part of the genome in a number of species, including mice, rats, monkeys, and humans [4], [5], [6], [7]. Comparison of the partial expression profiles of the aging mouse and human brain did not show significant overlap [8]. However, there has yet to be a systematic comparison of gene expression at a genome-wide scale in aging mice, monkeys, and humans. Recent advances in sequencing the rhesus macaque, mouse, and human genomes have enabled us to perform a genome-scale comparative analysis of gene expression in the aging mammalian brain [9], [10], [11], [12]. Although a small subset of age-related gene expression changes are conserved from mouse to man, major changes in the expression of genes involved in neurotransmission have evolved in the primate cortex that are potentially relevant to age-related changes in cognition and vulnerability to neurodegeneration.

## Results

### Phylogenetic Analysis of Brain Aging in Humans, Rhesus Macaques, and Mice

A central issue in a cross-species comparative analysis of aging is the identification of similar aging groups in species with very different maximal life spans. We previously defined the expression profile for age-related expression changes in the human cortex and demonstrated that these changes occur in the majority of individuals by the age of 70 years [6]. We used this expression profile as the basis for defining our aged group in humans as individuals older than 70 years of age who were not diagnosed with a neurodegenerative disorder (Table S1). To identify a similar age group in mice, we used comparative survival curves for humans and mice which suggest that a 30-month-old mouse is similar to an 81-year-old human since at these ages approximately 25% of the original populations survive. A similar survival analysis in rhesus monkeys in captivity determined that 25% survival occurred at approximately 26 years of age [13]. Hence, we chose 30 months and 28–31 years as the aged groups for mice and rhesus monkeys, respectively.

**Figure 1:**
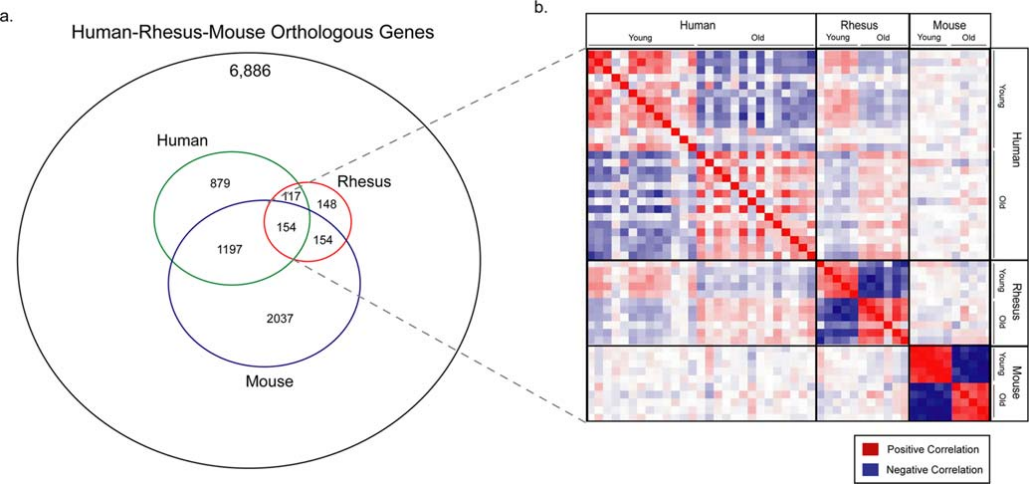
Genome-wide comparison of brain aging in humans, rhesus macaques, and mice. a. Venn diagram indicating the extent of overlap in age-related gene expression changes between the three species. The size of each circle corresponds to the number of age-related expression changes in each species. b. A group of 154 common aging genes provides an indicator of biological aging in all three species. Shown is a matrix of Pearson correlation coefficients that indicate the degree of overall similarity between any two samples (see Methods). Positively correlated sample pairs are indicated by red and negatively correlated pairs are indicated by blue. The degree of correlation correlates with color intensity. The species and age groups are indicated (Human: young ≤40 years; aged ≥70 years. Rhesus macaque: young 5–6 years; aged 28–31 years. Mouse: young 5 months; aged 30 months).

To identify age-related changes in gene expression, cortical samples from 13 young (≤40 years old) and 15 aged (≥70 years old) humans were hybridized to Affymetrix U133plus 2.0 arrays, 5-month-old (n = 5) and 30-month-old mice (n = 5) were hybridized to Affymetrix Mouse 430 2.0 arrays, and samples from 5–6-year-old (n = 5) and 28–31-year-old (n = 6) rhesus macaques were hybridized to Affymetrix whole genome rhesus arrays. Since the rhesus macaque genome has only recently been sequenced [9], the rhesus microarrays are based primarily on gene predictions. Therefore, we used an all-against-all protein BLAST to identify orthologous genes between the rhesus predictions and the other two species. For each homolog pair, we required a BLAST score of greater than 200, and at least 80% alignment of the human or mouse protein sequence with the rhesus sequence (Table S2). The final gene set was composed of genes that possess an ortholog in every species and are represented on all three array platforms. We then employed a two-sample t-test between young and aged age groups with a 1% false discovery rate (FDR) cut-off to identify 3542, 573, and 2347 age-related genes in mice, rhesus monkeys, and humans, respectively (Tables S3 [Supplementary-material pone.0003329.s008]–5). Among these age-related changes, only 154 were significantly associated with aging in all three species (Fig. 1a and Table S6). To assess this gene group as an indicator of brain aging, the behavior of all 154 genes was compared across age groups and species to derive Pearson correlation coefficients. The resulting correlation matrix showed that this core gene set distinguishes between young and aged samples in all three species (Fig. 1b). Furthermore, this set of age-related gene expression changes distinguished between chronological and biological age. For example, a 30-year-old rhesus monkey more closely resembled a 70-year-old human than a 30-year-old human. This set of common age related expression changes is therefore linked to the biology of the aging process in the brain.

**Figure 2:**
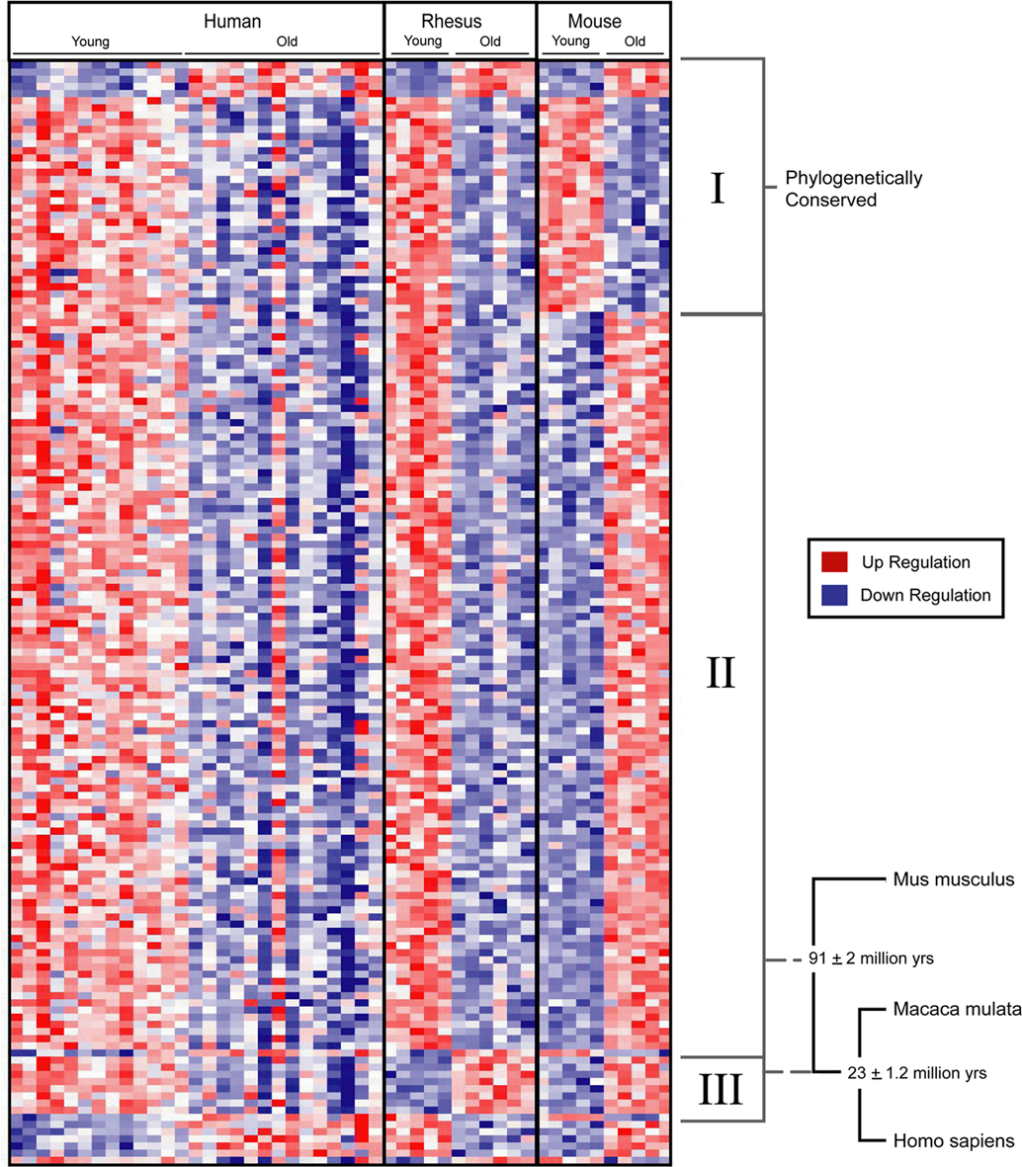
Age-regulated genes common to humans, rhesus macaques, and mice. The transcriptional profiles of genes that are age-regulated in all three species were analyzed by hierarchical clustering. Reduced expressed with aging is indicated by a transition from red in the young to blue in the aged, and vice versa. Genes separate into three groups based on whether the direction of age-related changes (i.e., age-upregulated or age-downregulated) is conserved in all three species (category I), changes between mice and rhesus macaques (category II), or changes between rhesus macaques and humans (category III). Also indicated is the evolutionary time of divergence in years for each pair of species based on analysis of protein sequence alignments [37].

Hierarchical clustering of the common age-regulated genes demonstrated that they fall into three distinct groups: I. Age-regulated genes that are conserved among all three species. II. Genes that change with age in all 3 species but differ in directionality between mouse and rhesus (e.g., from age-downregulated to age-upregulated); and III. Age-regulated genes that change directionality between rhesus and human (Fig. 2 and Table 1). Among the category I genes conserved in all 3 species, the most robustly age-upregulated gene was the anti-oxidant lipid binding protein apolipoprotein D. The most robustly age-downregulated genes in the conserved category were CAMK4, a component of synaptic cAMP-mediated signaling, and ARPP-21, a phosphoprotein also implicated in neuronal cAMP signaling [14] (Table 1). The genes in category II were composed almost entirely of genes that are age-upregulated in mice and downregulated in both rhesus monkeys and humans, defining a set of age-related gene expression changes common to rhesus monkeys and humans. The most robustly downregulated of these primate aging genes was calbindin 1 (CALB1), a marker of cortical inhibitory interneurons (Table 1).

**Table 1:** Age-regulated genes common to humans, rhesus macaques, and mice.

Gene Description	Gene Symbol	Q-Value (%)			Fold Change		
		Human	Rhesus	Mouse	Human	Rhesus	Mouse
**I. Phylogenetically Conserved Aging Genes**							
apolipoprotein D	APOD	0.035	0.856	0.017	2.251	4.006	2.245
G protein-coupled receptor, family C, group 5, member B	GPRC5B	0.279	0.232	0.017	1.642	1.412	1.415
tripeptidyl peptidase I	TPP1	0.390	0.438	0.017	1.496	1.247	1.145
ribosomal protein S9	RPS9	0.549	0.232	0.028	1.337	1.754	1.270
calnexin	CANX	0.090	0.232	0.057	1.200	1.371	1.677
solute carrier family 35 (UDP-galactose transporter), member A2	SLC35A2	0.035	0.438	0.028	−1.136	−1.525	−1.447
Cofactor required for Sp1 transcriptional activation, subunit 8, 34 kDa	CRSP8	0.195	0.856	0.120	−1.175	−1.323	−1.323
Hypothetical protein MGC29898	MGC29898	0.279	0.232	0.776	−1.195	−1.967	−1.306
glutathione synthetase	GSS	0.279	0.438	0.348	−1.200	−1.534	−1.603
ubiquitin-conjugating enzyme E2Q (putative) 1	UBE2Q1	0.549	0.720	0.639	−1.202	−1.534	−1.249
tRNA methyltranferase 12 homolog (S. cerevisiae)	TRMT12	0.740	0.501	0.240	−1.229	−1.452	−1.538
eukaryotic translation termination factor 1	ETF1	0.195	0.856	0.412	−1.231	−1.409	−1.215
hypothetical protein FLJ20232	RP5-1104E15.5	0.020	0.943	0.776	−1.244	−1.304	−1.283
dual specificity phosphatase 14	DUSP14	0.065	0.856	0.057	−1.260	−1.296	−1.428
member RAS oncogene family	RAB14	0.035	0.537	0.288	−1.294	−1.547	−1.314
Transmembrane protein 49	TMEM49	0.020	0.943	0.017	−1.299	−1.535	−1.613
NEDD8-conjugating enzyme	UBE2F	0.020	0.534	0.288	−1.300	−1.467	−1.119
zinc finger protein 64 homolog (mouse)	ZFP64	0.090	0.856	0.348	−1.304	−1.623	−1.508
transmembrane protein vezatin	VEZT	0.020	0.943	0.288	−1.318	−1.608	−1.293
transmembrane protein 4	TMEM4	0.020	0.537	0.120	−1.351	−2.788	−1.307
tribbles homolog 2 (Drosophila)	TRIB2	0.965	0.232	0.949	−1.365	−1.520	−1.209
Glutamine-fructose-6-phosphate transaminase 1	GFPT1	0.035	0.639	0.949	−1.385	−1.600	−1.923
protein disulfide isomerase family A, member 6	PDIA6	0.020	0.856	0.240	−1.436	−1.419	−1.303
Metallophosphoesterase domain containing 1	MPPED1	0.020	0.943	0.120	−1.441	−1.457	−1.307
armadillo repeat containing 8	ARMC8	0.195	0.438	0.057	−1.454	−1.472	−1.331
Ribonuclease H1	RNASEH1	0.020	0.599	0.120	−1.478	−1.425	−1.174
kelch repeat and BTB (POZ) domain containing 6	KBTBD6	0.020	0.537	0.057	−1.479	−1.548	−1.539
Membrane-associated ring finger (C3HC4) 1	MARCH1	0.195	0.639	0.120	−1.481	−1.361	−1.977
Acetoacetyl-CoA synthetase	AACS	0.020	0.775	0.412	−1.517	−1.347	−1.456
adrenergic, beta, receptor kinase 2	ADRBK2	0.020	0.856	0.057	−1.525	−2.077	−1.335
golgi autoantigen, golgin subfamily a, 1	GOLGA1	0.020	0.856	0.412	−1.565	−1.542	−1.192
transmembrane protein 14B	TMEM14B	0.020	0.438	0.039	−1.619	−1.876	−1.240
Transforming growth factor, beta receptor associated protein 1	TGFBRAP1	0.090	0.775	0.776	−1.738	−1.226	−1.117
Cyclic AMP-regulated phosphoprotein, 21 kD	ARPP-21	0.020	0.232	0.017	−1.953	−1.457	−1.137
Calcium/calmodulin-dependent protein kinase IV	CAMK4	0.020	0.438	0.193	−2.174	−2.111	−1.560
**II. Aging Genes that Diverged Between Mice and Rhesus Monkeys**							
calbindin 1, 28 kDa	CALB1	0.020	0.880	0.348	−3.722	−1.629	1.281
neuronal pentraxin II	NPTX2	0.020	0.880	0.017	−2.340	−1.815	1.323
chromobox homolog 6	CBX6	0.020	0.943	0.017	−2.305	−1.199	1.377
adenylate cyclase 2 (brain)	ADCY2	0.020	0.856	0.193	−1.973	−1.264	1.109
tubulin tyrosine ligase	TTL	0.020	0.599	0.057	−1.964	−1.527	1.198
3-hydroxy-3-methylglutaryl-Coenzyme A reductase	HMGCR	0.020	0.232	0.288	−1.866	−1.367	1.469
hepatic leukemia factor	HLF	0.020	0.639	0.017	−1.851	−1.250	1.337
similar to hepatocellular carcinoma-associated antigen HCA557b	LOC151194	0.020	0.501	0.240	−1.838	−1.479	1.127
trophoblast glycoprotein	TPBG	0.020	0.438	0.017	−1.809	−1.988	1.563
phospholipase C-like 2	PLCL2	0.020	0.375	0.017	−1.770	−1.566	1.163
Fusion (involved in t(12;16) in malignant liposarcoma)	FUS	0.020	0.959	0.412	−1.743	−1.535	1.145
protein phosphatase 3 (formerly 2B), catalytic subunit, beta isoform (calcineurin A beta)	PPP3CB	0.020	0.720	0.039	−1.725	−1.074	1.202
KIAA1944 protein	KIAA1944	0.020	0.232	0.949	−1.687	−1.673	1.169
Chromosome 18 open reading frame 1	C18orf1	0.020	0.537	0.949	−1.650	−1.299	1.331
phosphodiesterase 4D interacting protein (myomegalin)	PDE4DIP	0.195	0.534	0.057	−1.640	−1.333	1.292
similar to aspartate beta hydroxylase (ASPH)	ASPHD2	0.020	0.232	0.120	−1.628	−1.494	1.154
discs, large homolog 3 (neuroendocrine-dlg, Drosophila)	DLG3	0.020	0.880	0.085	−1.610	−1.228	1.124
adrenergic, alpha-2A-, receptor	ADRA2A	0.020	0.232	0.639	−1.597	−2.092	1.306
component of oligomeric golgi complex 8	COG8	0.020	0.775	0.017	−1.557	−1.327	1.274
protein tyrosine phosphatase, non-receptor type 3	PTPN3	0.020	0.856	0.949	−1.538	−1.701	1.201
Small nuclear ribonucleoprotein polypeptide A′	SNRPA1	0.020	0.943	0.146	−1.528	−1.217	1.250
RAS guanyl releasing protein 1 (calcium and DAG-regulated)	RASGRP1	0.020	0.232	0.098	−1.525	−1.648	1.150
Signal-induced proliferation-associated 1 like 2	SIPA1L2	0.020	0.232	0.500	−1.520	−2.293	1.189
Ubiquitin carboxyl-terminal hydrolase L5	UCHL5	0.020	0.775	0.028	−1.492	−1.999	1.357
neuregulin 3	NRG3	0.020	0.959	0.017	−1.491	−1.293	1.216
tubulin, alpha 1 (testis specific)	TUBA1	0.020	0.438	0.949	−1.486	−1.289	1.067
solute carrier family 36 (proton/amino acid symporter), member 1	SLC36A1	0.065	0.232	0.146	−1.482	−1.860	1.235
opsin 3 (encephalopsin, panopsin)	OPN3	0.020	0.943	0.057	−1.478	−1.430	1.268
bicaudal D homolog 2 (Drosophila)	BICD2	0.020	0.501	0.017	−1.470	−1.523	1.426
p21(CDKN1A)-activated kinase 7	PAK7	0.020	0.959	0.017	−1.455	−1.343	1.534
chromosome 21 open reading frame 5	DOPEY2	0.035	0.232	0.017	−1.453	−1.667	1.272
Nuclear factor I/B	NFIB	0.195	0.856	0.348	−1.450	−1.230	1.209
membrane associated guanylate kinase, WW and PDZ domain containing 1	MAGI1	0.020	0.639	0.500	−1.450	−1.364	1.167
TNF receptor-associated factor 3	TRAF3	0.279	0.232	0.776	−1.447	−1.498	1.112
small glutamine-rich tetratricopeptide repeat (TPR)-containing, beta	SGTB	0.090	0.959	0.017	−1.445	−1.806	1.316
hypothetical protein FLJ20701	FLJ20701	0.020	0.720	0.146	−1.442	−1.208	1.175
LanC lantibiotic synthetase component C-like 2 (bacterial)	LANCL2	0.020	0.639	0.639	−1.440	−1.380	1.113
Rho GTPase-activating protein	RICS	0.065	0.880	0.240	−1.437	−1.507	1.141
chromosome 10 open reading frame 9	C10orf9	0.020	0.856	0.017	−1.425	−1.379	1.490
dual-specificity tyrosine-(Y)-phosphorylation regulated kinase 2	DYRK2	0.195	0.232	0.039	−1.422	−1.821	1.251
Zinc finger protein 148 (pHZ-52)	ZNF148	0.020	0.599	0.017	−1.420	−1.336	1.320
similar to BcDNA∶GH11415 gene product	C3orf59	0.020	0.232	0.017	−1.412	−1.333	1.689
importin 11	IPO11	0.195	0.880	0.017	−1.408	−1.358	1.334
neuronal pentraxin receptor	NPTXR	0.090	0.943	0.288	−1.407	−1.199	1.310
solute carrier family 35, member B4	SLC35B4	0.020	0.537	0.017	−1.403	−1.780	1.341
secretogranin III	SCG3	0.020	0.720	0.500	−1.401	−1.255	1.125
Proprotein convertase subtilisin/kexin type 2	PCSK2	0.020	0.232	0.146	−1.399	−1.280	1.243
Programmed cell death 8 (apoptosis-inducing factor)	PDCD8	0.020	0.856	0.017	−1.397	−1.507	1.265
tripartite motif-containing 44	TRIM44	0.020	0.943	0.017	−1.387	−2.430	1.201
v-akt murine thymoma viral oncogene homolog 3 (protein kinase B, gamma)	AKT3	0.020	0.880	0.017	−1.385	−1.155	1.475
reticulon 4 receptor-like 1	RTN4RL1	0.020	0.959	0.288	−1.378	−1.369	1.187
WD repeat domain 32	WDR32	0.140	0.959	0.057	−1.378	−1.364	1.324
zinc finger, DHHC-type containing 4	ZDHHC4	0.020	0.639	0.146	−1.370	−1.995	1.181
karyopherin alpha 6 (importin alpha 7)	KPNA6	0.090	0.959	0.017	−1.362	−1.452	1.370
tribbles homolog 1 (Drosophila)	TRIB1	0.279	0.534	0.017	−1.359	−1.758	1.519
calmodulin regulated spectrin-associated protein 1	CAMSAP1	0.020	0.534	0.017	−1.351	−1.267	1.453
member RAS oncogene family	RAB22A	0.020	0.524	0.057	−1.345	−1.766	1.303
Calumenin	CALU	0.035	0.856	0.039	−1.344	−1.923	1.292
HEPIS	LOC119710	0.020	0.775	0.146	−1.342	−1.443	1.152
Ankyrin repeat domain 6	ANKRD6	0.140	0.943	0.098	−1.341	−1.361	1.220
kelch repeat and BTB (POZ) domain containing 7	KBTBD7	0.020	0.537	0.017	−1.327	−1.548	1.226
Rho-associated, coiled-coil containing protein kinase 2	ROCK2	0.020	0.232	0.120	−1.323	−1.773	1.289
dynactin 4 (p62)	DCTN4	0.090	0.375	0.028	−1.320	−1.306	1.317
UDP-glucuronate decarboxylase 1	UXS1	0.279	0.232	0.017	−1.315	−1.943	1.558
chromosome 1 open reading frame 21	C1orf21	0.020	0.599	0.017	−1.307	−1.326	1.259
proliferation-associated 2G4, 38 kDa	PA2G4	0.090	0.775	0.028	−1.303	−1.761	1.186
isocitrate dehydrogenase 2 (NADP+), mitochondrial	IDH2	0.965	0.720	0.057	−1.300	−1.538	1.292
ring finger protein 41	RNF41	0.140	0.375	0.057	−1.299	−2.333	1.286
ATPase, aminophospholipid transporter-like, Class I, type 8A, member 2	ATP8A2	0.035	0.720	0.949	−1.296	−2.771	1.272
zinc finger protein 697	ZNF697	0.279	0.524	0.057	−1.295	−1.543	1.193
makorin, ring finger protein, 1	MKRN1	0.020	0.537	0.500	−1.295	−1.224	1.085
eukaryotic translation initiation factor 3, subunit 12	EIF3S12	0.020	0.501	0.120	−1.294	−2.174	1.187
transforming, acidic coiled-coil containing protein 1	TACC1	0.035	0.537	0.017	−1.288	−1.945	1.968
THUMP domain containing 3	THUMPD3	0.090	0.639	0.098	−1.285	−1.648	1.175
mediator of RNA polymerase II transcription, subunit 8 homolog (yeast)	MED8	0.020	0.438	0.193	−1.282	−1.376	1.131
Casein kinase 2, alpha 1 polypeptide	CSNK2A1	0.020	0.959	0.098	−1.279	−1.367	1.572
metastasis associated 1 family, member 3	MTA3	0.390	0.959	0.017	−1.276	−1.299	1.268
DEAD (Asp-Glu-Ala-Asp) box polypeptide 54	DDX54	0.965	0.959	0.017	−1.272	−1.272	1.322
Ras-associated protein Rap1	RBJ	0.020	0.856	0.949	−1.270	−1.325	1.131
cleavage stimulation factor, 3′ pre-RNA, subunit 3, 77 kDa	CSTF3	0.020	0.534	0.146	−1.268	−1.145	1.472
N-myristoyltransferase 1	NMT1	0.090	0.943	0.017	−1.262	−1.174	1.340
Component of oligomeric golgi complex 1	COG1	0.035	0.959	0.017	−1.258	−1.685	1.659
SERPINE1 mRNA binding protein 1	SERBP1	0.035	0.943	0.240	−1.257	−1.303	1.143
kelch domain containing 3	KLHDC3	0.140	0.720	0.193	−1.252	−1.255	1.194
zinc finger protein 436	ZNF436	0.195	0.232	0.193	−1.249	−1.379	1.122
KIAA1217	KIAA1217	0.965	0.639	0.240	−1.247	−1.953	1.328
sideroflexin 4	SFXN4	0.020	0.639	0.639	−1.247	−1.233	1.182
ankyrin repeat domain 28	ANKRD28	0.065	0.856	0.120	−1.238	−1.505	1.345
Phosphodiesterase 8B	PDE8B	0.140	0.856	0.017	−1.236	−1.964	1.329
casein kinase 2, alpha prime polypeptide	CSNK2A2	0.020	0.943	0.288	−1.231	−1.315	1.137
Ras association (RalGDS/AF-6) domain family 5	RASSF5	0.965	0.524	0.017	−1.222	−1.416	1.304
microfibrillar-associated protein 1	MFAP1	0.090	0.501	0.017	−1.221	−1.347	1.280
tRNA nucleotidyl transferase, CCA-adding, 1	TRNT1	0.065	0.537	0.288	−1.219	−1.673	1.137
golgi SNAP receptor complex member 2	GOSR2	0.390	0.959	0.639	−1.216	−1.404	1.545
v-ral simian leukemia viral oncogene homolog A (ras related)	RALA	0.965	0.232	0.017	−1.207	−1.690	1.240
hypothetical protein FLJ11305	RP11-98F14.6	0.965	0.943	0.348	−1.205	−1.411	1.312
Zinc finger protein 291	ZNF291	0.279	0.943	0.017	−1.205	−1.342	1.458
UDP-N-acetyl-alpha-D-galactosamine (GalNAc-T2)	GALNT2	0.390	0.537	0.017	−1.202	−1.155	1.388
Acyl-Coenzyme A dehydrogenase family, member 9	ACAD9	0.020	0.232	0.017	−1.185	−1.364	1.403
deltex 4 homolog (Drosophila)	DTX4	0.020	0.599	0.017	−1.180	−1.254	1.314
casein kinase 1, gamma 1	CSNK1G1	0.279	0.856	0.017	−1.169	−1.430	1.274
KIAA1698 protein	KIAA1698	0.279	0.534	0.017	−1.157	−1.405	1.380
Yip1 domain family, member 3	YIPF3	0.965	0.599	0.017	−1.143	−1.703	1.540
Adducin 3 (gamma)	ADD3	0.065	0.599	0.085	1.540	1.329	−1.289
**III. Aging Genes that Diverged Between Rhesus Monkeys and Humans**							
cell division cycle 42 (GTP binding protein, 25 kDa)	CDC42	0.020	0.524	0.017	−1.688	1.608	1.186
melanoma antigen family H, 1	MAGEH1	0.020	0.232	0.776	−1.504	1.697	1.203
Seryl-tRNA synthetase	SARS	0.020	0.501	0.017	−1.453	1.375	1.650
clathrin, heavy polypeptide (Hc)	CLTC	0.020	0.375	0.057	−1.438	1.056	1.207
E-1 enzyme	MASA	0.020	0.524	0.949	−1.323	1.618	1.158
F-box protein 28	FBXO28	0.020	0.232	0.146	−1.301	1.701	1.155
abhydrolase domain containing 14A	ABHD14A	0.390	0.501	0.017	−1.192	1.318	1.404
eukaryotic translation initiation factor 1A, X-linked	EIF1AX	0.740	0.232	0.146	−1.175	2.762	1.278
hypothetical protein FLJ11155	FLJ11155	0.140	0.959	0.085	1.868	−3.539	−1.172

Shown are fold changes (aged to young intensity ratio; minus sign for age-downregulated, no sign for age-upregulated) and statistical q-values (%) derived by Significance Analysis of Microarrays (SAM) as described in Methods. Category I contains genes for which age-related expression changes are conserved, both in terms of significance and direction, across all three species. Category II contains genes in which the direction of the relationship with age changes from mouse to rhesus macaque. Category III contains genes in which the direction of the relationship with age changes between rhesus macaque and human.

### Cell-Type Enrichment of Age-Related Gene Expression Changes

To identify the cell types in the brain that exhibit prominent age-related changes in gene expression, we utilized the Allen Brain Atlas [15]. This database, derived by in situ hybridization and 3-dimensional imaging of the adult mouse brain (56 days old), includes genes in which expression was significantly enriched in one of five specific cell types. By combining this cell type analysis with our mouse gene expression data, a subset of age-related gene expression changes was localized to specific cell types. To determine whether the mouse brain dataset predicts the cell type distribution of these genes in the human brain, we performed microarray analysis of isolated neurons, astrocytes, and microglial cells derived from primary human cortical cultures, as previously described [16]. For each set of genes enriched in a specific cell type in mice, we determined the median fold enrichment in each of the three human cell type arrays. Genes that were predicted to be enriched in astrocytes and neurons in the mouse brain by the Allen database were also enriched in the corresponding cell types derived from the human cortex (Fig. S1 and Table S7).

**Figure 3:**
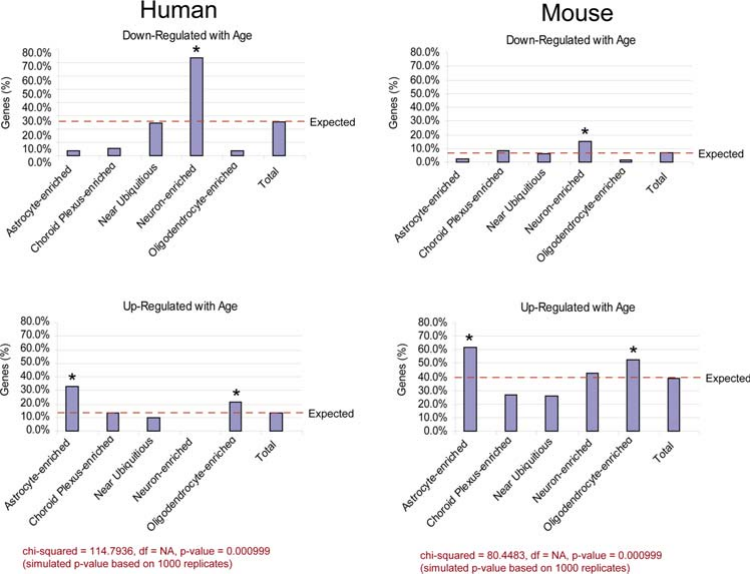
Cell type localization of gene expression in the aging cortex. Genes enriched in specific cortical cell types, based on the Allen Brain Atlas, were analyzed in the aging mouse and human gene expression profiles. The percentage of age-regulated genes enriched in each cell type is represented by the Y-axis was determined as described in Methods. The expected percentages are indicated by the dashed line. Statistically significant cell type enrichment was determined using a Chi-square test with a permutation-based p-value (1,000 replicates). Specific cell types that exhibit a statistically significant change in age-regulated genes are indicated by an asterisk.

The agreement between the human and mouse cell-type enrichment datasets enabled us to use the Allen Brain Atlas to localize age-related gene expression changes in both species. The limited number of age-related changes in the rhesus dataset prevented us from conducting a comprehensive cell type analysis in rhesus macaques. To determine whether there is a relationship between age-related expression changes and cell type localization, we determined the number of age-related gene expression changes that could be localized to each cell type using the Allen Brain Atlas. A relationship between age-related expression changes and cell type localization was analyzed statistically by determining if the number of age-regulated genes enriched in specific cell types deviated significantly from the number expected if these changes were independent of cell type localization. Both the human and mouse localization analysis showed significant deviation from values expected under the independence assumption (p-value<0.05). Statistical significance was assessed using a Chi-squared test in which the null distribution was estimated based on 1,000 replications (see Methods). The primary data and hypergeometric-based estimates are provided in Tables S8 and S9, respectively. Both humans and mice exhibit a larger fraction of age-upregulated astrocyte- and oligodendrocyte-enriched genes, and age-downregulated neuron-enriched genes, than would be expected by chance alone (Fig. 3). However, relative to mice, human aging is distinguished by a dramatic increase in the proportion of neuron-enriched downregulated genes (Fig. 3). This was also observed when the data was stratified by gender (Text S2). Analysis of our data using a different cell type transcriptome database, derived by isolation of astrocytes, neurons and oligodendrocytes from transgenic mouse cortex [17], confirmed that downregulation of neuronal genes distinguishes aging humans from aging mice (data not shown).

**Figure 4:**
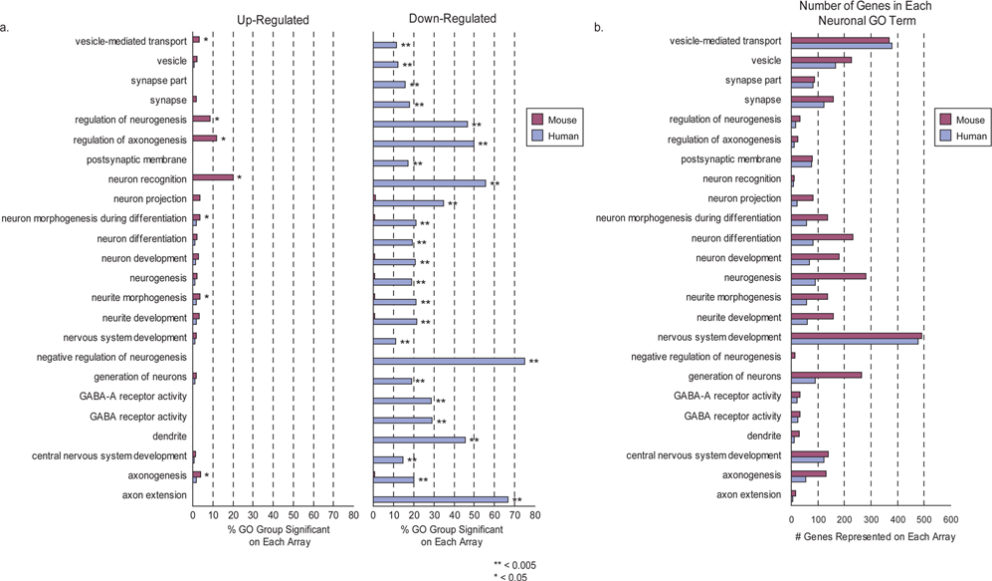
Neuronal gene ontology groups distinguish the expression profiles of the aging human and mouse cortex. a. Neuronal gene ontology (GO) groups that are significantly enriched (p-value≤0.005; binomial approximated p-value for a hypergeometric distribution) for age-related expression changes (SAM comparison, FDR≤0.01) were identified. The X-axis represents the percentage of genes in a GO group with age-related up- or down-regulation. Multiple neuronal GO groups are enriched in the human aging profile; while only a few neuronal GO terms appear at less significant thresholds in the mouse aging profile. Age-upregulated and age-downregulated genes are shown separately. b. Number of genes in each GO group that are represented on the mouse and human microarray platforms.

As an independent line of evidence for age-related downregulation of neuronal genes, we identified Gene Ontology (GO) groups that were significantly enriched for age-related expression changes (Table S10). In total, 24 neuronal GO groups were significantly enriched for age-related expression changes in humans (hypergeometric p-value<0.005) (Fig. 4a). In contrast, only 5 of these 24 neuronal GO terms were slightly enriched for genes significantly associated with age in mice (hypergeometric p-value<0.05), despite similar or greater gene numbers for each GO term represented on mouse versus human microarrays (Fig. 4b). Further characterization of these GO terms revealed that the vast majority of genes in the human-enriched neuronal GO terms were downregulated with age. In contrast, the significant mouse neuronal GO terms were primarily enriched for age-upregulated genes (Fig. 4a). Thus, aging reduces the expression of genes with a variety of neuronal functions to a much greater extent in humans than mice.

### Age-Related Repression of Genes Involved in Inhibitory Neurotransmission

**Figure 5:**
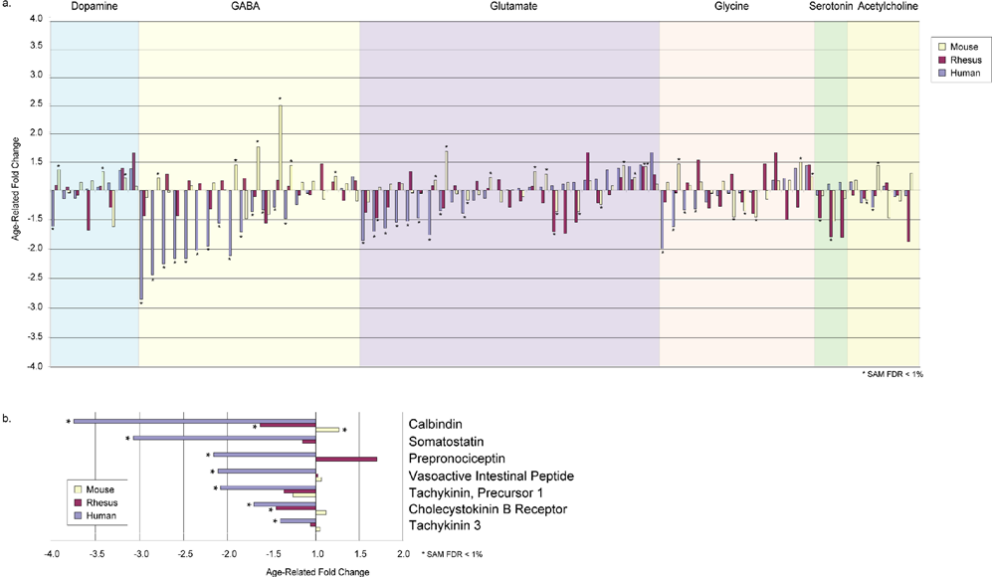
Global repression of genes associated with GABA-mediated inhibitory neurotransmission. Shown are age-related changes in the expression of genes that mediate major neurotransmitter systems in the cortex of humans, rhesus monkeys, and mice. a. Genes involved in specific neurotransmitters were identified based on membership in the corresponding GO groups. Age-related fold changes in genes with orthologs in all three species and represented on all three microarray platforms are shown for humans, rhesus monkeys, and mice. Gene identities are provided in Table S11. ^*^q-value≤0.01. b. Age-related fold changes for markers of inhibitory neuronal subpopulations. Statistical significance in a specific species (q-value≤0.01) is denoted with an asterisk.

Aging is associated with characteristic neurophysiologic and cognitive changes attributable to specific neurotransmitter systems. An important question, therefore, is whether age-related repression of neuronal genes selectively affects specific neurotransmitter systems. We noted that the only significantly enriched GO groups relating to a specific neurotransmitter were “GABA and GABA-A receptor activity” (Fig. 4). To explore this finding further, we examined the age-regulated expression of genes related to each of the major cortical neurotransmitters, including glutamate, gamma-aminobutyric acid (GABA), dopamine, glycine, serotonin, and acetylcholine (Fig. 5a). The most robustly age-regulated group corresponded to genes involved in GABA-mediated inhibitory neurotransmission (Fig. 5a and Table S11). Multiple genes in this category were age-downregulated with large fold changes in humans, including GABA A receptor subunits alpha 1 (GABRA1), alpha 5 (GABRA5), beta 3 (GABRB3) and gamma 2 (GABRG2), the GABA vesicular transporter (SLC32A1), and the GABA biosynthetic enzymes glutamate decarboxylase 1 and 2 (GAD1 and GAD2) (Table S11). Moreover, genes for the neuropeptides calbindin 1 (CALB1), somatostatin (SST), vasoactive intestinal peptide (VIP), cholecystokinin (CCK), tachykinin (TAC1), and nociceptin (PNOC), which are markers of inhibitory neuronal subpopulations in prefrontal cortex, were significantly age-downregulated (Fig. 5b). These genes were not significantly age-downregulated in mice, although some inhibitory markers, such as calbindin 1 and GABA A receptor subunit alpha 1, were significantly age-downregulated in rhesus macaques (Fig. 5). Downregulation of several glutamate-related genes, such as the glutamate receptor subunits AMPA 1 (GRIA1) and kainate 1 (GRIK1), was also observed, but the number and magnitude of these expression changes were less than that observed for GABA-related genes (Fig. 5a and Table S11). A subset of these age-related changes, notably calbindin 1, GABA A receptor subunit β3 and AMPA 1, have been confirmed by quantitative real time RT-PCR [6]. Thus, genes associated with inhibitory neurotransmission are repressed in the aging human cortex.

### Age-Related Reduction of Neuronal Proteins Is Not Associated with Overall Neuronal or Synaptic Loss

**Figure 6:**
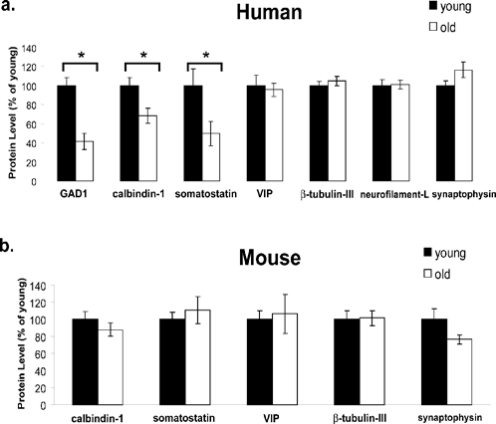
Reduced protein markers of inhibitory neurons in the aged human cortex. a. GAD1, calbindin-1, and somatostatin protein levels are significantly lower in the aged (71–91 yr; white) human cortex than in the young adult (24–35 yr; black) cortex, in agreement with microarray results. VIP expression is age-stable at the protein level. The neuronal markers β-tubulin-III and neurofilament-L are age-stable at the protein level, as is the synaptic protein synaptophysin. n = 15. The primary Western blot data are shown in Figure S2a. b. Calbindin-1, somatostatin, and VIP protein levels are age-stable in the mouse cortex, in agreement with the microarray results. Likewise, β-tubulin-III and synaptophysin do not change significantly with age. Attempts to probe for mouse GAD1 and neurofilament-L were not successful. n = 6. The primary Western blot data are shown in Figure S2b. In both a and b, the level of each protein was normalized to the level of actin. Values represent the mean±S.E.M. expressed as percent of the mean young value for each protein. * P<0.05 by Student's two-tailed t-test.

To determine whether reduced mRNA levels are associated with reduced protein levels in the aging brain, a subset of gene products expressed in GABAergic neurons was examined by quantitative Western blotting in cortical samples from young adult and aged humans and mice. The protein level of the major GABA biosynthetic enzyme in the brain, GAD1, was significantly reduced in the aging human cortex, as well as the levels of calbindin 1 and somatostatin, in agreement with the microarray data (Fig. 6a and Fig. S2a). The neuropeptide VIP did not show a significant age-related change at the protein level, in contrast to the age-related reduction in VIP mRNA. This difference may reflect limited sensitivity of the antibody used for Western blotting of VIP, or post-translational regulation of VIP levels. In contrast to aging human cortex, the aging mouse cortex did not exhibit altered levels of calbindin or somatostatin, which is also in agreement with the microarray data (Fig. 6b and Fig. S2b).

Stereological cell counting studies suggest that neuronal loss is not significant in the aging human prefrontal cortex. To confirm this finding, we performed quantitative Western blotting for two established neuron-specific markers, β-tubulin III and neurofilament L chain [17]. The levels of both proteins did not change significantly in the aging human prefrontal cortex (Fig. 6a and Fig. S2a). We also examined the presynaptic marker synaptophysin, which did not show a significant age-related change in this cortical region (Fig. 6a and Fig. S2a). These results suggest that downregulation of neuronal genes in the aging human cortex cannot be attributed to overall loss of neurons or synapses.

## Discussion

We have compared the protein-coding transcriptome of the aging cerebral cortex in mice, rhesus monkeys, and humans by utilizing species-specific genome-scale microarrays. As such, this study is not confounded by cross-species hybridization of RNA to microarrays, and provides a broad view of the evolution of the mammalian aging brain. Our results suggest that a relatively small subset of age-regulated gene expression changes are conserved from mouse to man. The most robustly age-upregulated of these conserved genes is apolipoprotein D, which has been shown to protect against oxidative stress and extend lifespan in Drosophila [18], [19]. Moreover, apolipoprotein D is upregulated at the protein level in the aging human brain and to a greater extent in a variety of neurological diseases, including Alzheimer's disease [20], [21]. The most robustly age-downregulated gene conserved in all three species is CAMK4, a key component of the cAMP signaling cascade that links synaptic activity to CREB-dependent transcription and modulates synaptic plasticity [14], [22]. Another key cAMP signaling gene, adenylate cyclase 2, is age-downregulated in humans and rhesus macaques. Thus, increased expression of neuroprotective genes and reduced expression of genes involved in synaptic function are conserved features of mammalian aging.

Localization of gene expression by in situ hybridization and analysis of gene ontology groups indicates that 3 cell types – astrocytes, oligodendrocytes, and neurons – exhibit significant age-dependent changes in gene expression in mice and humans. However, age-related downregulation of neuronal genes has increased dramatically from mouse to man, and is a major distinguishing feature. Several lines of evidence suggest that this is unlikely to be secondary to neuronal cell death. First, stereological analysis of neuronal cell number did not detect neuronal loss in the region of the aging human prefrontal cortex used in this study [2], [23]. Second, we have shown that expression of a number of neuron-specific genes is unaltered in the aging human prefrontal cortex at both the mRNA and protein levels. Moreover, the absence of a significant age-related change in synaptophysin levels suggests that overall synapse numbers may also be preserved. However, this does not rule out more subtle changes in synaptic or dendritic spine structure as reported in aging rhesus monkeys [24]. Finally, we showed previously that age-related gene downregulation did not correlate with postmortem interval in the range used in our study [6], consistent with the lack of an effect of postmortem interval on RNA integrity in another study [25]. In addition, we monitored brain tissue pH to exclude human cases with prolonged terminal hypoxia [26]. Taken together, these findings are consistent with a primary age-related change in the regulation of neuronal gene expression. In a previous study, we found that downregulated neuronal genes were associated with DNA damage in the aging human cortex, and that DNA damage can repress the transcription of these genes in primary neuronal cultures [6]. Another study suggested that some genes undergo age-dependent DNA methylation [27]. Thus, transcriptional repression in neurons may be a primary feature of human brain aging that has evolved in long-lived primates.

A systematic investigation of genes involved in the major cortical neurotransmitter systems suggests that the GABA system, which mediates inhibitory neurotransmission, may be particularly affected in the aging human prefrontal cortex. This is underscored by the 50–60% reduction in mRNA and protein levels of GAD1, the primary GABA biosynthetic enzyme in the brain. In addition, the marked downregulation of calbindin 1 and somatostatin suggests that specific inhibitory neuronal subpopulations may be unusually vulnerable. Reduced calbindin 1 immunocytochemical staining has also been demonstrated during normal brain aging in rhesus monkeys and humans, and becomes more pronounced in Alzheimer's disease [28]. Thus, aging of the brain may be associated with reduced inhibitory neurotransmission.

The central role of GABA in cognition and affective state raises the possibility that age-dependent downregulation of this system might contribute to neurophysiological and psychological changes in the aging population [29]. Reduced inhibitory circuit activity might increase cortical activation during the performance of routine cognitive tasks, a phenomenon that has been demonstrated in the aging human prefrontal cortex by functional imaging studies [30], [31]. This pattern of increased cortical activation may initially be compensatory, enabling aged individuals to function at a higher level [31]. However, increased excitation could predispose to excitotoxicity, a mechanism of neuronal cell death associated with a variety of age-related neurological disorders, including Alzheimer's disease [32]. Functional imaging studies have implicated cortical overactivation due to impaired inhibitory function in patients with Alzheimer's disease [33]. The relevance of overexcitation to disease pathogenesis is suggested by the clinical efficacy of the NMDA receptor antagonist memantine, currently the only treatment that delays progression of moderate to late stage Alzheimer's disease [34]. Interestingly, significant downregulation of GABA-related genes is not detected in the aging mouse cortex, which may increase resistance to excitotoxicity relative to aging humans. This may, in turn, contribute to the paucity of neuronal cell death in mouse models of neurodegenerative diseases compared with the human pathology [2]. Hence, a greater understanding of normal brain aging and its evolution may provide new insights into pathogenic mechanisms involved in age-related neurodegeneration.

## Materials and Methods

### Samples and Microarray Platforms

All aspects of animal housing and experimental procedures were approved by the Institutional Animal Care and Use Committees of Children's Hospital Boston and the Beth Israel-Deaconess Hospital (for rhesus macaques) and by the William S. Middleton V.A. Medical Center and the University of Wisconsin-Madison Medical School (for mice). Postmortem human tissue was procured in accordance with institutional guidelines. Detailed description of the human, rhesus macaque and murine samples and extraction protocols are supplied in Text S1 and Table S1. Postmortem human cortical samples were derived from subjects that did not carry a diagnosis of Alzheimer's disease or another neurodegenerative disease, and showed neuropathological findings within the normal range for age. In addition, human brain tissue samples with a pH>6.5 were used to exclude prolonged terminal hypoxia [26]. We generated genome-wide expression profiles of young and aged cortical samples in humans, rhesus monkeys and C57BL/6J mice using Affymetrix Human Genome U133plus 2.0 arrays, Rhesus Macaque Genome arrays and Mouse Genome 430 2.0 arrays, respectively. Affymetrix Human Genome U133plus 2.0 arrays were also used for expression profiling of neurons, microglia and astrocytes isolated from primary fetal human cortical cultures. Samples with acceptable parameters of RNA quality (Text S1) were hybridized to the corresponding Affymetrix oligonucleotide arrays, which were then scanned and expression data extracted using the standard Affymetrix Microarray Suite Software.

### Gene Mapping

Predicted rhesus macaque proteins, based on the Jan. 2006 version of the rhesus genome (Baylor College of Medicine HGSC v1.0), were aligned to human Refseq protein sequences mapped to NCBI Build 36 of the human genome. The mapping was conducted using the BLAST program [35] by first creating a BLAST protein database from the predicted rhesus proteins. Using protein-BLAST, individual human Refseq proteins were then compared to the rhesus protein database. A BLAST score greater than 200, and at least 80% of the human protein aligning to the predicted rhesus protein, was required to declare an orthologous pair between the two species. A complete list of orthologous human-rhesus gene pairs is provided in Table S2.

### Expression Profiling and Analytical Approaches

To compare expression across species, genes were required to have orthologs in the human, rhesus macaque and mouse genome databases, and to have probesets in the microarray platforms for each species. We also required that probes be called present using dChip software in at least 20% of the arrays for each species. Four approaches were used to analyze the genes meeting these criteria. **1.** Significance Analysis of Microarrays (SAM) software was used to compare young and aged groups within each species with the following criteria for identifying age-related expression changes: 1000 permutations and median false discovery rate (FDR) ≤0.01. Significant age-related gene expression changes are listed for humans, rhesus macaques and mice in Tables S3, S4, and S5, respectively. The subset of genes that are age-regulated in all three species is provided in Table S6. These common age-related genes were also resolved by hierarchical clustering using dChip software (build date: April 11, 2007) [36]. The display range used in the hierarchical clustering was 2.0 (a value greater than 2.0 standard deviations above the mean is pure red, below is pure blue, and equal to the mean is white). **2.** Correlation coefficients between samples were calculated and visualized using dChip software across the 154 genes that are significantly associated with aging in all three species. The range of observed correlation coefficients was (−0.78, 0.82), excluding a sample's correlation with itself. The display range used was 0.7 (correlation above 0.7 is pure red, below is pure blue, and 0 is white). **3.** The cell type enrichment analysis of age-regulated genes was performed independently for mice and humans, and included all genes that met the above microarray criteria and were also present in the list of cell type-enriched genes in the Allen Brain Atlas (Table S8). For the analysis in humans, we required that the mouse ortholog to the human gene be present in the list. For each species, the number of significant age up- and down-regulated genes, as well as the number of non-significant genes, was determined for each of the five cell types indicated in the Allen Brain Atlas resulting in a 3-by-5 table (Table S9). Assuming independence between cell types and age-related expression changes, the expected count within each cell of the table was estimated using the row and column totals. As a result of the low count in some cells, a Monte Carlo p-value, based on 1000 replications was calculated for each species to test whether the observed count significantly deviated from what was expected by chance. **4.** Gene Ontology analysis was performed independently for humans and mice using dChip software (Table S10). A Gene Ontology group is considered to be enriched in the aging database if it contains a greater number of significantly age-related genes than expected by chance. The statistical significance of GO group enrichment is determined using a binomial approximation to the hypergeometric distribution with a p-value cut-off of 0.005, as described in detail elsewhere (www.dchip.org) [36].

### Analysis of Cultured Human Cortical Cell Types

Neurons, astrocytes and microglia were isolated from primary fetal human cortical cultures as described previously [16]. Expression profiling of the isolated cortical cell types was performed using Affymetrix U133 plus 2.0 arrays. To assess the concordance of gene expression profiles of corresponding human and mouse cortical cell types, we analyzed the probe sets on the human U133plus 2.0 arrays that corresponded to the Allen Brain Atlas list of mouse cell type-enriched genes (Table S7). Fold enrichment of a particular gene in a specific human cell type was calculated as follows: the intensity of the gene in one cell type was divided by the maximum of the intensities in the two remaining cell types. The median fold enrichment of a particular human cell type was then calculated over all of the genes that were called enriched in a specific mouse cell type (Allen Brain Atlas). The result was a human cell type enrichment score for every human-mouse cell type combination (Fig. S1). Median fold values greater than 1.0 indicated enrichment.

### Western Blot Analysis

#### Human Brain

Postmortem human cortical tissue (Brodmann area 9/10) was flash frozen and stored at −150° until use. Tissue was homogenized in RIPA-DOC buffer containing protease inhibitors (Complete, Roche) with microcystin (1 µm) and Na_3_Vo_4_ (1 mM). Tissue was homogenized, sonicated, and centrifuged at 10,000 rpm at 4°C and the protein concentration in the resulting supernatant was assayed (BioRad protein assay). Samples were boiled in 1× SDS sample buffer containing DTT and resolved by 7% SDS-PAGE using the Criterion System (BioRad) and electrotranferred to PVDF membranes (Immobilon, Millipore). The primary antibodies and dilutions used to probe the PVDF membranes are described in Table S12. Secondary antibodies (Jackson ImmunoResearch) were used at 1∶2000 diluted in 5% nonfat milk. Blots were developed on film or with a gel documentation system (Syngene) and quantified with GeneTools software (Syngene).

#### Mouse Brain

Three young B6C3F1 mice (5 months) and three aged B6C3F1 mice (30 months) were sacrificed and the cortex was isolated and homogenized in 20 mM HEPES, 125 mM NaCl, 0.1% NP40, 0.1% Triton X-100, 1 mM EDTA, 10 mM nicotinamide, 1 µM trichostatin A, protease inhibitors (Complete, Roche), and phosphatase inhibitors (PhosSTOP, Roche). Samples were boiled in SDS sample buffer containing DTT, resolved by SDS-PAGE on 10% or 12.5% Tris-glycine gels and electrotransferred to PVDF membranes.

## Supporting Information

Text S1 The MIAME Checklist
(0.07 MB DOC)

Text S2 Analysis of the Effects of Gender
(0.03 MB DOC)

Figure S1 Concordance of Gene Expression in Human and Murine Neural Cell Types
(0.63 MB TIF)

Figure S2 Western Blot Analysis of Age-Downregulated and Age-Stable Proteins in Human and Mouse Cortex.
(8.39 MB TIF)

Table S1 Sample information
(0.03 MB XLS)

Table S2 Mapping of predicted rhesus macaque and human genes
(6.28 MB XLS)

Table S3 Age-Related Expresssion Changes in Humans
(1.16 MB XLS)

Table S4 Age-Related Expression Changes in Rhesus Macaques
(0.42 MB XLS)

Table S5 Age-Related Expresssion Changes in Mice
(1.78 MB XLS)

Table S6 Genes with Significant Age-Related Expression Changes in Humans, Rhesus Macaques, and Mice
(0.08 MB XLS)

Table S7 Concordant Expression Patterns in Cultured Human Cortical Cell Types and the Allen Brain Atlas
(0.07 MB XLS)

Table S8 Age-Related Expression Changes of Genes Enriched in Specific Cortical Cell Types In Vivo
(0.12 MB XLS)

Table S9 Calculations for Determining Significant Cell-Type Enrichment of Age-Regulated Genes
(0.03 MB XLS)

Table S10 Gene Ontology Enrichment Analysis of Human and Mouse Expression Data
(0.05 MB XLS)

Table S11 Age-Related Gene Expression Changes Classified by Neurotransmitter Type
(0.05 MB XLS)

Table S12 Antibodies employed in the analysis of cortical protein levels
(0.02 MB XLS)
