# Supplementary material for: Evolution of the Aging Brain Transcriptome and Synaptic Regulation
Source: PLoS One. 2008 Oct 2;3(10):e3329. doi: 10.1371/journal.pone.0003329 (PMC2553198; doi:10.1371/journal.pone.0003329)
Supplement: Text S1 — The MIAME Checklist (0.07 MB DOC) [file pone.0003329.s001.doc]

**Text S1. The MIAME Checklist**

**Experimental Design**

**1. Type of Experiments**

**I.** Comparison of the gene expression profiles of young and old cortical brain samples in humans, rhesus monkeys, and mice. Young and old ages within each species and number of samples within each group are specified in #6 below.

**II**. Comparison gene expression profiles of neuron, microglia, and astrocytes human progenitor cells

**2. Experimental factors**

**I.** Age, Species

**II.** Cell Type

**3. Number of hybridizations performed**

**I.** 28 hybridizations on Affymetrix HG-U133plus_2 chips, 11 hybridizations on Affymetrix whole

genome rhesus chips, and 10 hybridizations on Affymetrix Mouse430_2 chip.

**II.** 3 hybridizations on Affymetrix HG-U133plus_2 chips

**4. Hybridization design**

**I.** Comparison of 13 young and 15 aged human brain samples, comparison of 5 young and 6 old rhesus monkey brain samples, and comparison of 5 young and 5 aged c57BL/6J mouse brain samples.

**II.** Comparison of microglia, neuron, and astrocyte human progenitor samples

**5. Quality control steps taken**

Only samples that met Affymetrix empirical quality control criteria (e.g., GAPDH 3’:5’ ratio < 2.5) were chosen for hybridization onto any of the Affymetrix platforms.

**6. Number of replicates**

**I.** Human: 13 young (≤40 yr) and 15 aged (≥70 yr) prefrontal cortex samples were analyzed in the group comparison

Rhesus Monkey: 5 young (5-6 yr) and 6 aged (28-31 yr) prefrontal cortex samples were analyzed in the group comparison

Mouse: 5 young (5 months) and 5 aged (30 months) neocortex samples from B6C3 Fi hybrid mice were analyzed in the group comparison

**II.** Neuron, microglia, and neuron samples were each analyzed on a single chip

**7. URL and any supplemental websites or database accession numbers**

All primary CEL files will be placed on a supplemental website prior to publication

**Samples, Preparation, and Labeling**

**1. Biological samples**

**Humans:** The postmortem brain tissue samples were neuropathologically normal for age, and were derived from non-demented individuals. Tissue was procured in accordance with institutional guidelines. Human cortical grey matter samples were dissected from the frontal pole (Brodmann area 9, 10), and were snap frozen in liquid nitrogen and stored at -85ºC. Human brain samples with a tissue pH > 6.5 were used to exclude prolonged terminal hypoxia [1].

**Rhesus Monkeys:** Rhesus monkeys were euthanized according to institutionally approved protocols. For rhesus monkeys, the cortex forming the principalis gyrus extending to the upper and lower rami of the arcuate sulcus was dissected as this region corresponds to the prefrontal cortex (area 9/46) in humans [2]. Samples were snap frozen in liquid nitrogen and stored at -85ºC.

**Mouse:** Male B6C3 Fi hybrid mice were purchased from Harlan Sprague-Dawley at 6-7 weeks of age. Mice were housed singly under specific pathogen-free conditions in the AAALAC-approved Shared Aging Rodent Facility at the William S. Middleton V.A. Medical Center. Mice were provided acidified water ad libitum and were fed 98 kcal/week of AIN-93M diet, which is ~90% of the average ad libitum food intake of these mice, on a schedule of 2X their daily food allocation on Mondays and Wednesdays and 3X their daily allocation on Fridays. Mice were euthanized at 5 and 30 months of age by rapid cervical dislocation and autopsied to exclude animals showing overt disease. A brain region equivalent to the human prefrontal cortex has not been established in mouse, for this reason and because the amount of cortical tissue is limited in mouse, the neocortex was dissected away from the cerebellum, hippocampus and other sub-cortical tissues and immediately flash-frozen in liquid nitrogen. Tissues were stored at -80°C until preparation of RNA for microarray analysis. All aspects of animal housing and experimental procedures were approved by the Institutional Animal Care and Use Committees of both the William S. Middleton V.A. Medical Center and the University of Wisconsin-Madison Medical School.

**Human Cortical Cells:** Primary human fetal cortical cultures were established, and astrocytes, neurons, and microglia were isolated by fluorescence activated cell sorting using cell type-specific markers, as described previously [3].

**2. RNA Isolation and Processing**

**Human and Rhesus Monkey:** Dissected cortical grey mater was cut into small pieces in the frozen states and ~70 mg was homogenized immediately in Trizol (Gibco) and RNA was isolated. RNA that was intact by electrophoresis and had an A_260_/A_280_ ratio ≥ 1.9 was used for cDNA synthesis. cDNA synthesis, cRNA synthesis and fragmentation, and preparation of the hybridization cocktail were carried out according to the Affymetrix protocol (Affymetrix GeneChip Expression Analysis Technical Manual). Sample information is provided in Table S1.

**Mouse:** Total RNA was extracted from tissues using the TRIzol reagent (Invitrogen, Carlsbad, CA) Polyadenylate [poly(A+)] RNA was purified from the total RNA with oligo-dT-linked Oligotex resin (Qiagen, Santa Clarita, CA). One microgram of poly(A+) RNA was converted into double-stranded cDNA (ds-cDNA) by using the SuperScript ChoiceSystem (Invitrogen) with an oligo-dT primer containing a T7 RNA polymerase promoter (Genset, La Jolla, CA). The ds-cDNA was extracted with phenol-chloroform-isoamyl alcohol and aqueous and organic phases were separated using phase lock gel (Brinkmann Instruments, Westbury, NY). The ds-cDNA was recovered from the aqueous by precipitation with ethanol and the co-precipitant pellet paint (Novagen, Madison, WI). Biotin-labeled RNA was synthesized using the Bioarray high-yield RNA transcript labeling kit (Enzo, Farmingdale, NY). The biotin-labeled antisense cRNA was purified using the RNeasy affinity column (Qiagen) and fragmented randomly to sizes ranging from 35 to 200 base pairs by heating to 94°C for 35 min. Preparation of the hybridization cocktail was carried out according to the Affymetrix protocol (Affymetrix GeneChip Expression Analysis Technical Manual). Sample information is provided in Table S1.

**Human Cortical Cells:** Cells were extracted in Trizol (Gibco) and RNA was isolated. RNA that was intact by electrophoresis and had an A_260_/A_280_ ratio > 1.9 was used for cDNA synthesis. cDNA synthesis, cRNA synthesis and fragmentation, and preparation of the hybridization cocktail were carried out according to the Affymetrix protocol (Affymetrix GeneChip Expression Analysis Technical Manual).

**3. External Controls (spikes)**

Affymetrix standard external spikes (BioB, BioC, BioD and Crex) were added to hybridization cocktail at 1.5, 5, 25, and 100 pM, respectively.

**Hybridization Procedures and Parameters**

**1. The protocol and conditions used during hybridization, blocking and washing**

Hybridizations were performed in the Genechip hybridization over 640 (60vrpm) for 16 hrs at 45ºC. The probe arrays were then washed, stained in the GeneChip Fluidics Station 400 operated by GeneChip software following the appropriate fluidics protocols.

**Measurement Data and Specifications**

**1. The quantization based on the images**

Original Affymetrix.dat output files

**2. The set of quantitations from multiple arrays upon which the authors base their conclusions**

- **Type of scanning and image analysis hardware and software**

The Microarray Suite Software controlled HP G2500A GeneArray Scanner was utilized to scan the surface of probe arrays and the converted digital intensity values were stored as image data files (*.dat) and then further converted into cell intensity files (*.cel) using Affymetrix Microarray Suite 5.0.

- **A description of the measurements produced by the image-analysis software and a description of which, measurements were used in this analysis**

Affymetrix Microarray Suite 5.0 was utilized to generate Affymetrix present/absent calls. The dChip software (build date: April 11, 2007, [www.dchip.org](http://www.dchip.org/), [4] was used to independently normalize the arrays for each species and the progenitor cells at probe level and compute model-based expression values using the PM/MM model.

- **The complete output of the image analysis before data selection and transformation**

The primary CEL files are provided

- **Data selection and transformation procedures**

**I.** Genes were required to have a homologous gene in humans, rhesus monkeys and mice, as well as be represented on the array platforms used in each species. We also required that every probe was called ‘present’ by dChip software on at least 20% of the arrays within each species. Significance Analysis of Microarrays (SAM) software [5] was then used to compare the young and aged groups within each species with the following criteria: 1000 permutations and median false discovery rate (FDR) < 0.01. A list of statistically significant age-related genes was generated for each species (Tables S3-5). dChip software was used to produce the hierarchical clustering of the 154 genes that are significantly associated with age in all three species (Fig. 2, Table 1). The correlation coefficients between samples (Fig. 1b) were calculated and visualized using dChip software across the 154 genes that are significantly associated with aging in all three species. In the cell-type enrichment analysis (Fig. 3), the additional restriction that genes be contained in the Allen Brain Atlas [6] list of cell type enriched genes was also applied. All bar charts (Figs. 3-5) were originally produced using Microsoft Excel and the Excel graphs and data are provided in Tables S8-11.

**II.** We required that every probe corresponded to genes that were contained in the Allen Brain Atlas list of cell type enriched genes. Fold enrichment of a particular gene in a specific human cell type was calculated as follows: the intensity of the gene in one cell type was divided by the maximum of the intensities in the two remaining cell types. The median fold enrichment of a particular human cell type was then calculated over all of the genes that were called enriched in a specific mouse cell type (Allen Brain Atlas). The result was a human cell type enrichment score for every human-mouse cell type combination. Median fold enrichment values greater than 1.0 were considered to be an indication of enrichment. The raw data, transformed data, and original Excel graphs are provided in Table S7.

- **Final gene expression data tables**

Provided in Table 1 and Tables S3-6.

**Array Design**

**1. Platform Type**

Synthesized Oligonucleotide Array

**2. Surface and Coating Specifications**

Glass

**3. Availability of the Array**

Human: Affymetrix Human Genome U133plus 2.0 Array

Rhesus Monkey: Affymetrix Rhesus Macaque Genome Array

Mouse: Affymetrix Mouse Genome 430 2.0 Array

Human Progenitor Cells: Affymetrix Human Genome U133plus 2.0 Array

**4. For each feature (spot) on the array, its location on the array and the ID of its respective reporter (molecule present on each spot)**

Human Genome U133plus 2.0 Array <http://www.affymetrix.com/products/arrays/specific/hgu133plus.affx>

Rhesus Macaque Genome Array:

<http://www.affymetrix.com/products/arrays/specific/rhesus_macaque.affx>

Mouse Genome 430 2.0 Array:

<http://www.affymetrix.com/products/arrays/specific/mouse430_2.affx>

**5. For each reporter, its type (e.g., cDNA or oligonucleotide) should be given, along with information that characterizes the reporter molecule unambiguously, in the form of appropriate database reference(s) and sequence (if available)**

Human Genome U133plus 2.0 Array <http://www.affymetrix.com/products/arrays/specific/hgu133plus.affx>

Rhesus Macaque Genome Array:

<http://www.affymetrix.com/products/arrays/specific/rhesus_macaque.affx>

Mouse Genome 430 2.0 Array:

<http://www.affymetrix.com/products/arrays/specific/mouse430_2.affx>

**References**

1. Kingsbury AE, Foster OJ, Nisbet AP, Cairns N, Bray L, et al. (1995) Tissue pH as an indicator of mRNA preservation in human post-mortem brain. Brain Res Mol Brain Res 28: 311-318.

2. Petrides M, Pandya DN (1999) Dorsolateral prefrontal cortex: comparative cytoarchitectonic analysis in the human and the macaque brain and corticocortical connection patterns. Eur J Neurosci 11: 1011-1036.

3. Wang J, Gabuzda D (2006) Reconstitution of human immunodeficiency virus-induced neurodegeneration using isolated populations of human neurons, astrocytes, and microglia and neuroprotection mediated by insulin-like growth factors. J Neurovirol 12: 472-491.

4. Li C, Wong WH (2001) Model-based analysis of oligonucleotide arrays: expression index computation and outlier detection. Proc Natl Acad Sci U S A 98: 31-36.

5. Tusher VG, Tibshirani R, Chu G (2001) Significance analysis of microarrays applied to the ionizing radiation response. Proc Natl Acad Sci U S A 98: 5116-5121.

6. Lein ES, Hawrylycz MJ, Ao N, Ayres M, Bensinger A, et al. (2007) Genome-wide atlas of gene expression in the adult mouse brain. Nature 445: 168-176.
