# Supplementary material for: Evolution of the Aging Brain Transcriptome and Synaptic Regulation
Source: PLoS One. 2008 Oct 2;3(10):e3329. doi: 10.1371/journal.pone.0003329 (PMC2553198; doi:10.1371/journal.pone.0003329)
Supplement: Text S2 — Analysis of the Effects of Gender (0.03 MB DOC) [file pone.0003329.s002.doc]

**Text S2. Analysis of the Effects of Gender**

The availability of samples limited our study to the use of only male mice and only female rhesus monkeys. However, the human dataset was approximately evenly divided between males and females with 6 young men, 7 young women, 8 aged men, and 7 aged women. The large number of both male and female human samples allowed us to address the possibility of gender differences in age-related expression changes. To accomplish this, we calculated the young-old, t-statistic for every gene that was present on at least 20% of the human arrays, independently for men and women. The gender-specific t-statistics and fold-changes between young and aged individuals were significantly correlated between males and females (0.88 and 0.93, respectively), suggesting that there is no significant gender difference. In addition, we repeated the cell-type enrichment analysis independently for males and females. Significance Analysis of Microarrays (SAM) was used to identify expression changes that were significantly associated with age (FDR ≤ 0.01) within each gender. Separating the male and female samples reduces the sample size to approximately half of the original analysis. As a result of this reduction in sample size, the total percentage of significant genes enriched for each cell type was less for both males and females when compared to the combined analysis. However, in agreement with the combined analysis, both males and females showed approximately three times more age-downregulated, neuron-enriched genes than expected by chance. Males also showed upregulation of oligodendrocyte- and choroid plexus genes, which was not detected in women. However, this enrichment was based on only five genes and is therefore tentative. In addition to overall downregulation of neuron-enriched genes, the age-related decline of genes involved in inhibitory neurotransmission, in particular specific subunits of the GABA-A and GABA-B receptors, also remains when the data are analyzed within each gender. These results suggest that the set of human age-regulated genes is highly concordant between males and females. Moreover, the human-specific downregulation of neuron-enriched genes, and in particular GABA-related genes, does not show a gender-specific difference.
