# Supplementary figures and images for: Evolution of the Aging Brain Transcriptome and Synaptic Regulation
Source: PLoS One. 2008 Oct 2;3(10):e3329. doi: 10.1371/journal.pone.0003329 (PMC2553198; doi:10.1371/journal.pone.0003329)

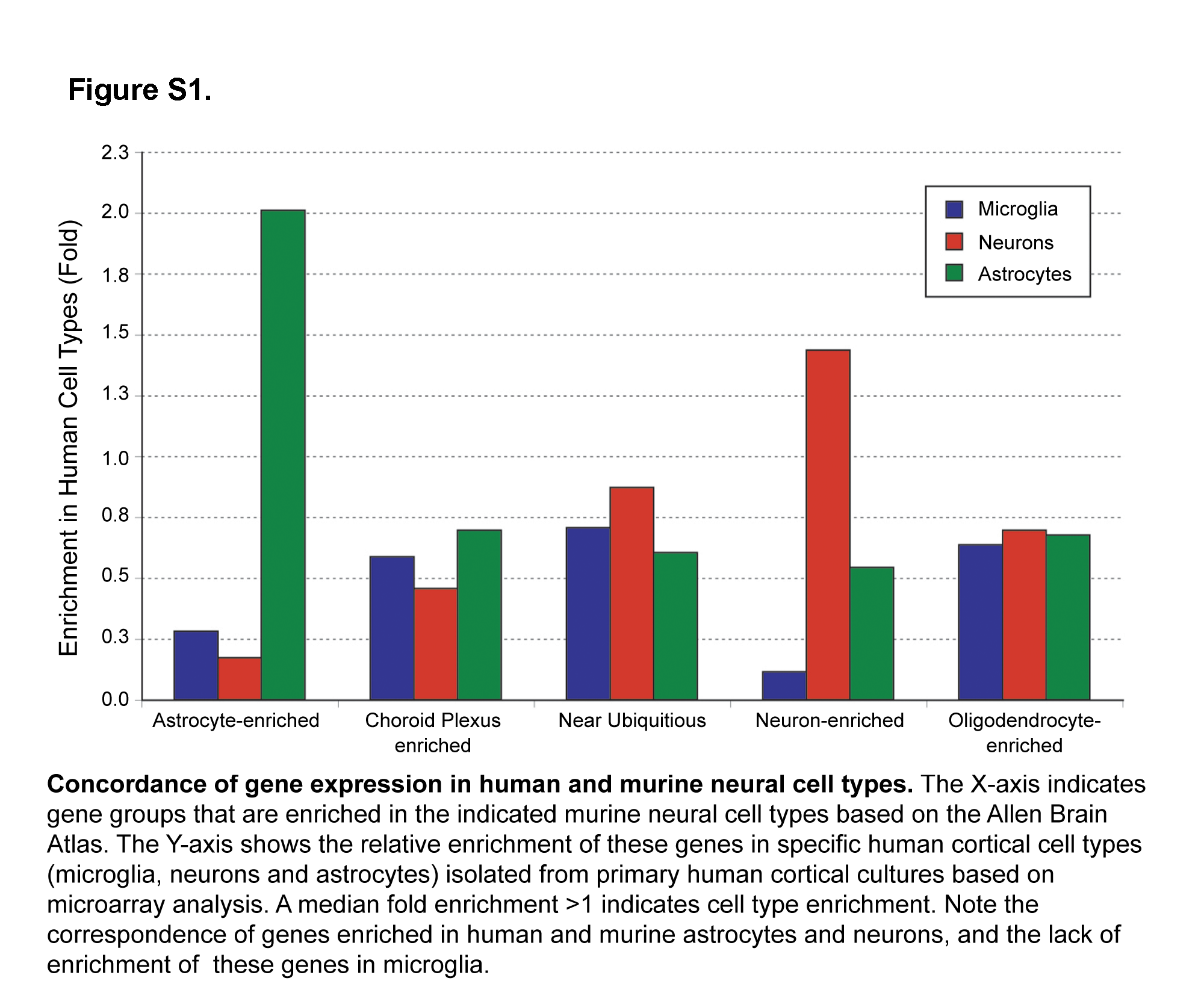

Supplement: Figure S1 — Concordance of Gene Expression in Human and Murine Neural Cell Types (0.63 MB TIF) [file pone.0003329.s003.tif]

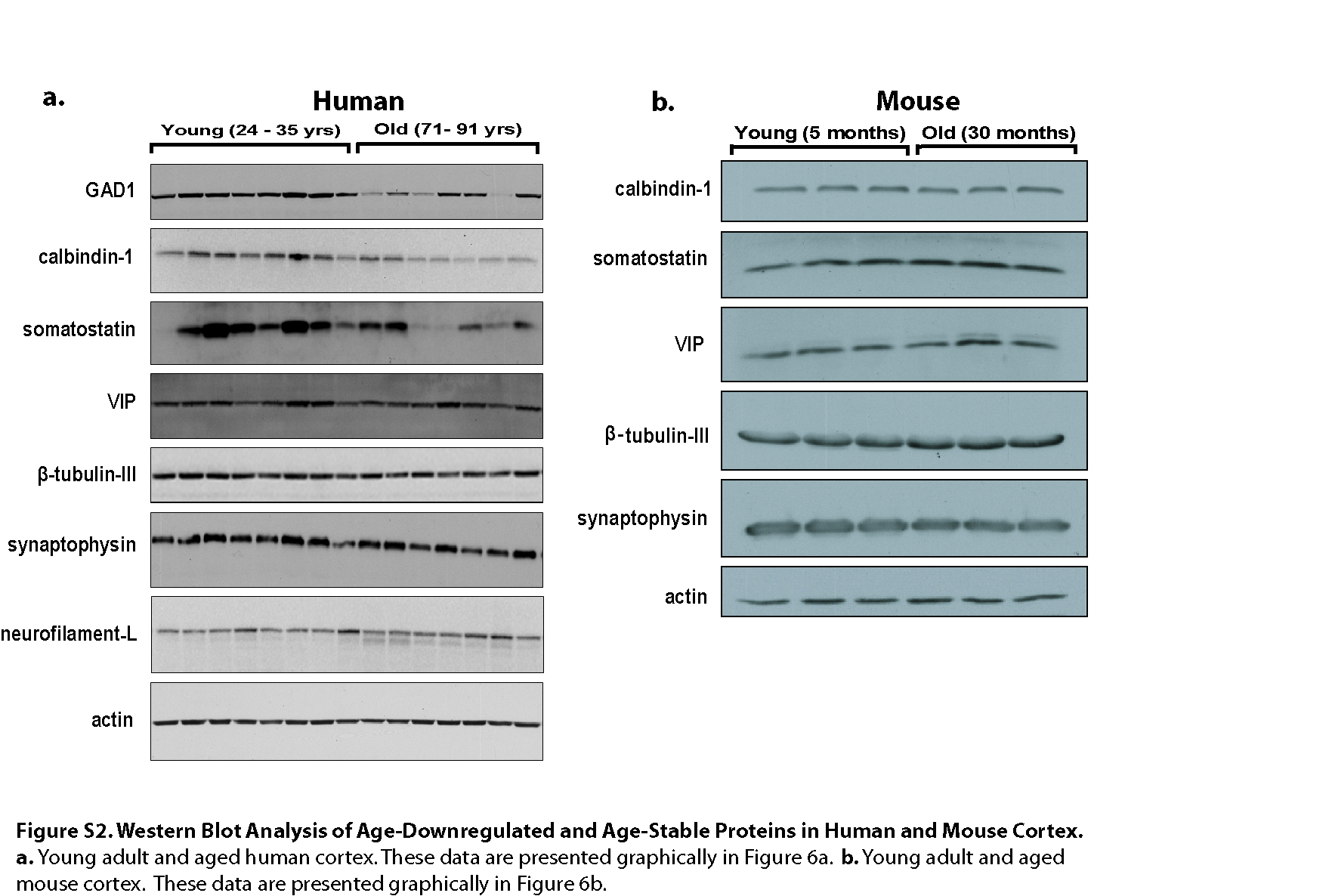

Supplement: Figure S2 — Western Blot Analysis of Age-Downregulated and Age-Stable Proteins in Human and Mouse Cortex. (8.39 MB TIF) [file pone.0003329.s004.tif]
